# Evidence that Life History Characteristics of Wild Birds Influence Infection and Exposure to Influenza A Viruses

**DOI:** 10.1371/journal.pone.0057614

**Published:** 2013-03-04

**Authors:** Craig R. Ely, Jeffrey S. Hall, Joel A. Schmutz, John M. Pearce, John Terenzi, James S. Sedinger, Hon S. Ip

**Affiliations:** 1 U.S. Geological Survey, Alaska Science Center, Anchorage, Alaska, United States of America; 2 U.S. Geological Survey, National Wildlife Health Center, Madison, Wisconsin, United States of America; 3 J. S. Sedinger, Department of Natural Resources and Environmental Science, University of Nevada, Reno, Nevada, United States of America; University of Georgia, United States of America

## Abstract

We report on life history characteristics, temporal, and age-related effects influencing the frequency of occurrence of avian influenza (AI) viruses in four species of migratory geese breeding on the Yukon-Kuskokwim Delta, Alaska. Emperor geese (*Chen canagica*), cackling geese (*Branta hutchinsii*), greater white-fronted geese (*Anser albifrons*), and black brant (*Branta bernicla*), were all tested for active infection of AI viruses upon arrival in early May, during nesting in June, and while molting in July and August, 2006–2010 (*n* = 14,323). Additionally, prior exposure to AI viruses was assessed via prevalence of antibodies from sera samples collected during late summer in 2009 and 2010. Results suggest that geese are uncommonly infected by low pathogenic AI viruses while in Alaska. The percent of birds actively shedding AI viruses varied annually, and was highest in 2006 and 2010 (1–3%) and lowest in 2007, 2008, and 2009 (<0.70%). Contrary to findings in ducks, the highest incidence of infected birds was in late spring when birds first arrived from staging and wintering areas. Despite low prevalence, most geese were previously exposed to AI viruses, as indicated by high levels of seroprevalence during late summer (47%–96% across species; *n = 541*). Seroprevalence was >95% for emperor geese, a species that spends part of its life cycle in Asia and is endemic to Alaska and the Bering Sea region, compared to 40–60% for the other three species, whose entire life cycles are within the western hemisphere. Birds <45 days of age showed little past exposure to AI viruses, although antibodies were detected in samples from 5-week old birds in 2009. Seroprevalence of known age black brant revealed that no birds <4 years old had seroconverted, compared to 49% of birds ≥4 years of age.

## Introduction

Avian influenza (AI) viruses (*Orthomyxoviridae*), are found in many species of birds throughout the world, especially waterfowl (Family Anatidae). Continued outbreaks of highly pathogenic avian influenza (HPAI) H5N1 viruses, and the associated threat of a pandemic, have sustained worldwide interest in AI viruses [1], [2]. Although HPAI H5N1 has not yet been detected in North America, pathogenic strains of AI viruses virulent to poultry are not uncommon, and in the Pacific Flyway exemplified by an outbreak in the spring of 2004 when more than 17 million domestic birds were culled in British Columbia [3]. As pathogenic strains of AI viruses are known to generate from avirulent forms found in wild birds [4], it is imperative to better understand the natural history of low pathogenic (LP) AI viruses to help avert future impacts of AI viruses on local economies and human health [5], [6].

Species-specific characteristics of host organisms have been implicated as likely predictors of transmission rates of AI viruses, although few such studies have been conducted [5], [7], [8]. Social factors such as gregariousness, vagility, site fidelity, dispersal characteristics, and habitat preferences, may all influence viral exposure. Such behavioral characteristics constitute a key subset of life history attributes, under the common definition of life history as “a set of evolved strategies, including behavioral, physiological and anatomical adaptations, that more or less influence survival and reproductive success directly” [9]. Indeed, social indices such as degree of aggregation have been used for predicting viral movement among waterfowl in Europe [10], and AI virus infection in ducks has been shown to be highest in late summer when birds congregate in large flocks and immunologically naïve young comprise a large part of the sample [7]. More specifically, modeling and predicting future AI virus outbreaks will require a detailed understanding of natural host populations and the environments they utilize, including species differences “related to general behavior, spatial and temporal distribution, habitat utilization, migration behavior, population age structure, and individual species susceptibility” [6]. One way to investigate the influence of species ecology on viral dynamics is to study a suite of sympatric host species with different life history characteristics, which to date has not been done in a major serological investigation of infection dynamics in a suite of species.

Alaska is the primary breeding area in the Nearctic for many species of waterfowl, and because it is at the apex of several international flyways, is likely a mixing area for birds and pathogens [11], [12], [13]. Furthermore, western Alaska is a region with a high proportion of AI viruses with foreign origin gene segments [13]. The Yukon-Kuskokwim Delta in western Alaska is a relic of Beringia; and because of proximity, and strong traditional trans-Beringian avian migration routes, a region strongly implicated in intercontinental viral connections [11], [12], [14], [15]. Previous work in Alaska showed that few bird species in Alaska in general, and geese in particular, are infected with low pathogenic AI viruses (1.7% for all species, <3% for geese; [14]). One possible conclusion from this finding might be that geese breeding in Alaska are rarely infected by AI viruses. However an alternative explanation is that geese have a higher incidence of exposure to AI viruses during other periods of their annual cycle.

Most work on AI viruses in wild birds has focused on testing for viral prevalence, and simple monitoring has been the primary goal of HPAI virus surveillance programs [10], [14], [16]. Surveillance for birds infected with HPAI viruses has provided valuable information not only for monitoring HPAI virus prevalence, but also for identifying the diversity of host species and geographic extent of low pathogenic forms of the AI virus. However, at any given time only a portion of a focal species is likely to be infected with the virus, which makes it difficult to identify species that carry AI viruses through standard swab testing [17], especially if the duration of viral shedding in very short [18]. This problem can be addressed with serological testing, and recent investigations of the long-term exposure of birds to AI viruses have been very informative in revealing species differences in exposure [19], [20], and seasonal variation in antibody levels within a species [21]. Serological information is also important as past exposure to LPAI viruses may provide protection from future AI virus infection, even across subtypes [22], [23], [24], [25], [26]. Therefore, knowledge of historical exposure in populations should improve our understanding of population immunity and provide insights into interpreting degree of infection and the dynamics of virus transmission. Serological studies are also invaluable for assessing the importance of ecological and life history parameters of host species on viral ecology [5], [27]. Such investigations should be based on species and population differences in viral exposure, not simply viral prevalence, because the latter can vary across seasons and years, and estimates of viral contact might therefore be misleading if target organisms are only sampled for a single season.

**Figure 1:**
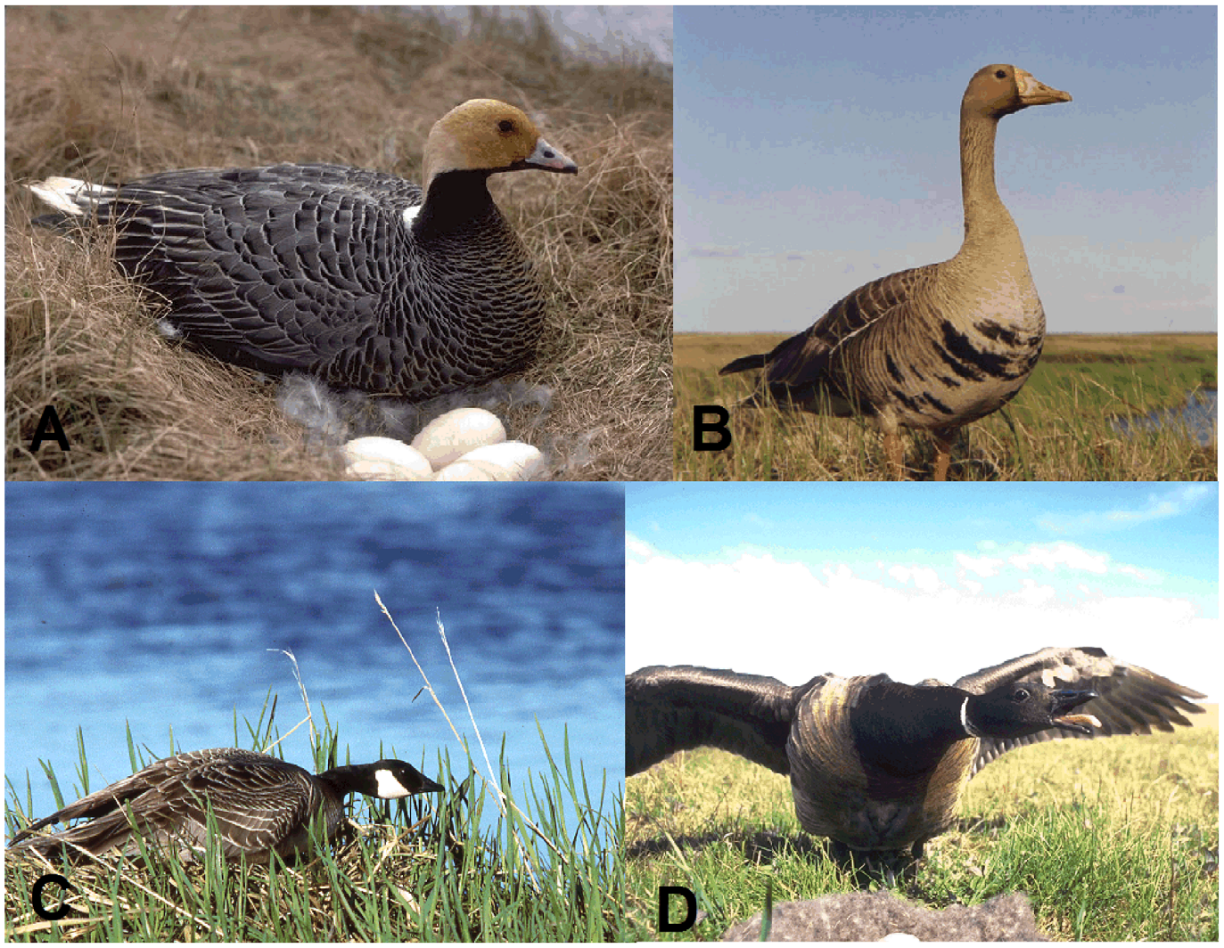
Photographs of the four sympatric nesting species of geese studied in the investigation on the Yukon-Kuskokwim Delta, Alaska: A) Emperor goose (*Chen canagica*), B) Greater white-fronted goose (*Anser albifrons*), C) Cackling goose (*Branta hutchinsii*), and D) Black brant (*Branta bernicla*).

To determine whether the results from our assessment of AI virus infection in Alaska during summer [14], are representative of overall population exposure we investigated the prevalence of antibodies in four species of geese breeding in western Alaska; species for which we have extensive knowledge of natural history, behavioral ecology and population genetic structure. Our study is the first to simultaneously investigate the prevalence of influenza A antibodies and AI virus prevalence across a group of sympatric-nesting waterfowl species. We report on differences in the frequency of occurrence of AI viruses in four species of sympatric breeding geese, the emperor goose (*Chen canagica*), cackling goose (*Branta hutchinsii*), greater white-fronted goose (*Anser albifrons*), and black brant (*Branta bernicla*) (Figure 1). Although co-occurring during the breeding season, these species exhibit pronounced differences in life history traits, such as social behavior, winter habitat use and distribution, molt migration, and continental affinities (Table 1).

**Table 1:** General life history traits among four sympatrically breeding geese in western Alaska examined in this study.

Life history parameter	Black brant	Emperor goose	Cackling goose	Greater white-fronted goose
Social index	Colonial nesting	Dispersed nesting	Dispersed nesting	Dispersed nesting
Winter habitat	Marine	Marine	Agricultural	Agricultural
Staging and winter distribution	Pacific Coastal Lagoons	Aleutian Islands, AK	Interior valleys of WA, OR, CA	Interior valleys of WA, OR, CA
Molt migration	Lengthy	Lengthy	Near breeding area	Near breeding area
Continental affinity	Nearctic	Nearctic and Palearctic	Nearctic	Nearctic

## Materials and Methods

### Ethics Statement

This study was approved by the Institutional Animal Care and Use Committees (IACUC) of the U.S. Fish and Wildlife Service Region 7, and the U.S. Geological Survey (USGS), Alaska Science Center (ASC), under Federal Permit #s MB124771 (2006) and MB789758 (2007–2010), and ASC IACUC permit # 2008–15 (2009–2010). The study was also approved by the IACUC of the University of Nevada Reno (protocol 00056). Blood samples were also collected under the authority of Federal Bird Banding Permit #20022 from the U.S. Department of Interior.

### Study Area and Sampling Chronology

**Figure 2:**
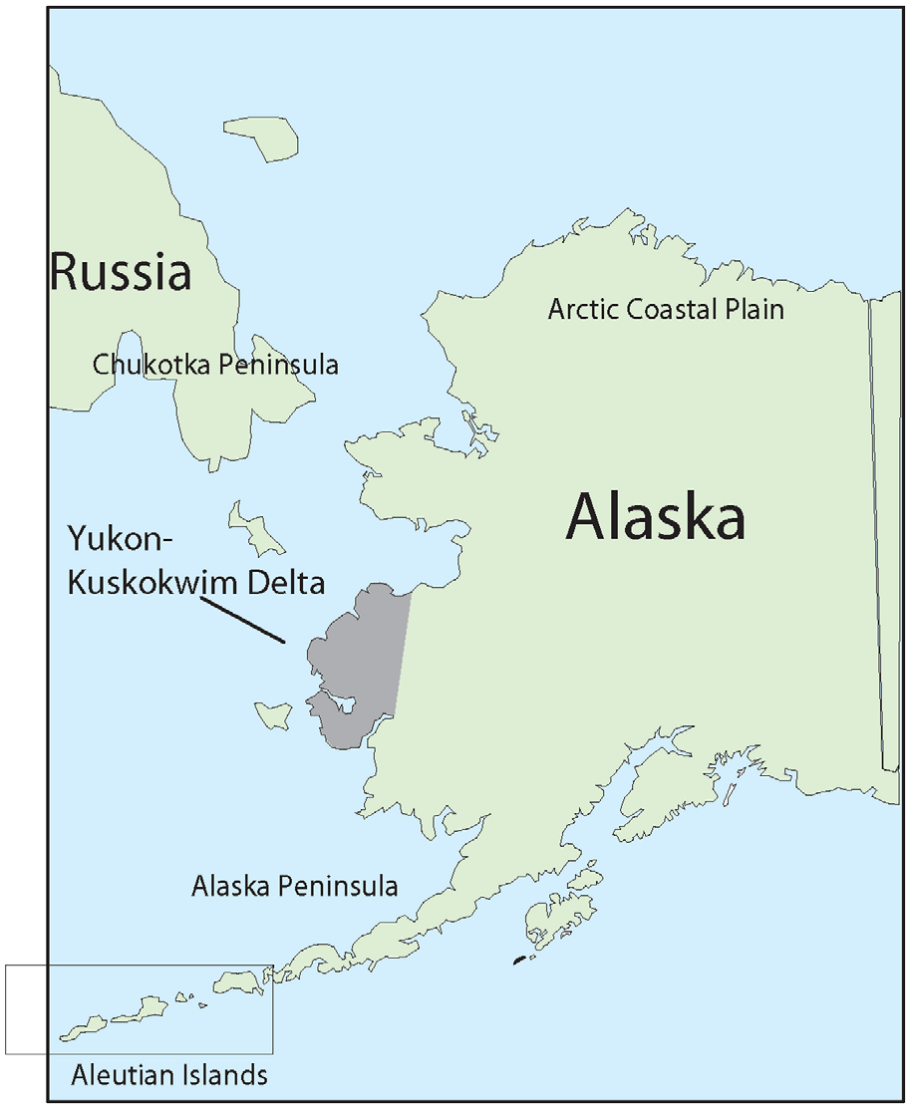
Location of sampling of geese on the Yukon-Kuskokwim Delta, Alaska, 2006–2010, relative to the location of staging and molting areas used by the different species of geese.

Samples were obtained from all four goose species on the Yukon-Kuskokwim Delta, Alaska, May–August, 2006–2010 (Figure 2). Geese tested for active AI virus infection were sampled upon their arrival in early May, during nesting (June) and again in late July and early August, 2006–2010 before regaining flight and migrating south. Geese tested for past exposure to AI viruses were sampled only during the period of wing molt in late July and early August, 2009–2010.

### Sample Acquisition

Samples collected during May were from birds harvested by subsistence hunters in the vicinity of villages along the coast of the Bering Sea coast of the Yukon-Kuskokwim Delta, Alaska. Small-bodied Canada geese (*Branta canadensis*) are difficult to distinguish from cackling geese, and although the spring samples were obtained within the breeding range of cackling geese, it is possible some of the samples are of Canada geese, which have a more interior distribution in Alaska [28]. Subsequent samples (June–August) were from live-captured geese. Methods of live capture and handling procedures followed protocols established by the USGS Alaska Science Center, and the University of Nevada Reno Animal Care and Use Committee. For nesting birds this consisted of trapping incubating females, and for molting geese entailed herding flightless birds into drive traps. Molting geese were captured when goslings were approximately 4–6 weeks of age. Geese were classified to sex based on cloacal examination and determination of age (adult or hatch year) was based on plumage characteristics [29]. In 2009 and 2010 we selectively targeted previously marked black brant during captures to obtain a sample of known-age birds, ranging from 1–16 years old. Since marked birds were more likely to be females (due to male dispersal), sex ratios were not 50: 50.

### Antibody Detection

Samples for detecting antibody prevalence were obtained by collecting approximately 4 ml of blood from each goose via jugular venipuncture. The blood was then transferred to vacutainers containing a separating matrix (BD Vacutainer® reference 367986). We waited for blood to clot for several hours before centrifuging at 3000 rpm, or until the supernatant was clear. Serum was removed from the top of the vacutainers and transferred to cryovials before transporting from the field in liquid nitrogen shippers. Serum samples were tested for antibodies to influenza A viruses with a commercial enzyme-linked immunosorbent assay (ELISA) FlockChek®, AI virus Antibody Test Kit (IDEXX Laboratories Inc., Westbrook, Maine, USA) as per manufacturer’s instructions. The IDEXX ELISA tests for influenza A nucleoprotein antibodies. The ELISA test is designed to detect prior exposure to all subtypes of AI viruses, as it detects antibodies to the conserved nucleoprotein of the influenza A virus, and has been shown to be effective for a wide variety of avian species [20], [30], [31], [32].

### Detection of AI Viruses

Active shedding of AI viruses was determined using real time reverse transcriptase polymerase chain reaction (RT-PCR) for molecular detection of viruses in swab samples according to the standardized USDA National Animal Health Laboratory Network Avian Influenza Protocol [14], [33], [34], [35]. Cloacal swabs were collected in 2006, whereas in 2007–2010 cloacal and oral-pharyngeal (OP) swabs were obtained and either pooled in the field or the laboratory. Swab samples were analyzed individually or pooled in the laboratory in groups of 2 to 5 by sampling location and species and detection of AI viruses was determined according to the standardized USDA National Animal Health Laboratory Network AI (RT-PCR) protocol [36], [37]. Individual samples in positive pools were then re-tested by matrix RT-PCR to identify the specific sample or samples that were AI virus positive. Follow-up tests were not conducted on OP swabs. All matrix RT-PCR test positive specimens, as well as a percent of negative specimens, were further tested by virus isolation in embryonating eggs [14], however, because of differences in determining the prevalence of AI viruses using RT-PCR versus viral isolation [14], we restricted our detection of AI viruses to RT-PCR to make our findings more comparable to other studies [38], [39]. Differences in AI virus prevalence have also been found depending on which part of a bird is sampled, with a higher incidence of LPAI viruses being found in oral samples versus cloacal samples in greater white-fronted geese [40], [41], although the reverse has been reported for other species of waterfowl [36], [40], [42].

### Statistical Analysis

Our target sample for each species at each location was 200 individuals, a number determined necessary to detect AI viruses at a prevalence of 1.5% in the focal population [37]. We used logistic regression (PROC LOGISTIC; [43]) to compare variation in prevalence of AI virus infection relative to species and year of sampling. We also used logistic regression to model factors influencing the prevalence of AI antibodies in adult geese. Covariates in candidate models included: 1) Species, 2) AI Virus Prevalence (proportion of individuals testing positive for AI viruses via RT-PCR testing in spring), 3) Social Index (colonial or dispersed nesting), 4) Continental Affinity (solely North America or including Asia), 5) Wintering Habitat (marine or agriculture), and 6) Staging and Wintering Distribution (isolated or co-occurring). We did not include molt migration as a model parameter as groupings were identical to Winter Habitat. We included year, sex, and sex+year with each of the covariates in the model, which lead to a total of 28 models considered (including the null model). We modeled the influence of age on seroprevalence of known age black brant using logistic regression as well. In all cases, we used Akaike’s Information Criteria (AIC) to guide model selection [44]. From the best fitting model, we estimated the proportion that had been infected (for each group) as simply the number that tested positive divided by the total number sampled. We calculated 95% Clopper-Pearson exact confidence intervals [45] around these proportions rather than the more typical confidence intervals derived from asymptotic standard errors [46], [47] because the latter is incalculable when the proportion is zero. The Clopper-Pearson confidence intervals are somewhat conservative and are nearly always slightly larger than other methods of confidence interval calculation [47].

## Results

### Prevalence of AI Infection

**Figure 3:**
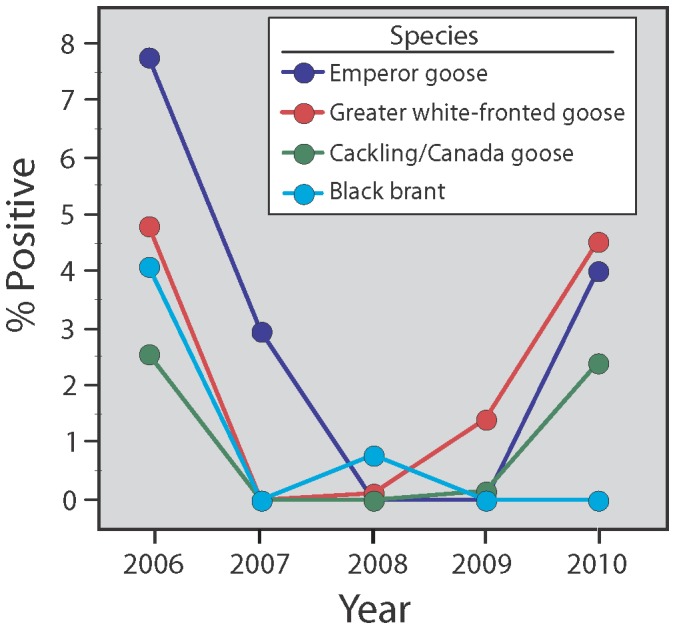
Prevalence of low pathogenic avian influenza viruses in subsistence-harvested geese during spring on the Yukon-Kuskokwim Delta, Alaska, 2006–2010. (See Table 1 for sample sizes). Data for 2006 previously presented [14].

Few geese tested positive for LPAI viruses, as RT-PCR results revealed that less than 1% of the 14,323 geese sampled were actively shedding viral RNA across the three sampling periods (Table 2, Table S1). LPAI virus detection was most common during spring (1.78%, *n = *8076), and least common during the period of wing molt in late summer (0.0%, *n* = 5192). Only 2 of 1055 (0.19%) incubating females tested positive for AI virus RNA. The percent of birds actively shedding viruses in spring varied annually and across species, as the best model included year and species (Table 3, Figure 3). There was some support for a model that included a species effect and year represented as a quadratic function where the middle years of study had a lower incidence of AI virus infection. AI virus Infection during spring was highest in 2006 (4.80% across 4 species) and 2010 (2.73%), and lowest in 2007, 2008, and 2009 (0.73, 0.22, and 0.39% respectively). Overall, the incidence of AI viruses in spring was 35% higher in emperor geese than greater white-fronted geese (2.94±1.44% vs. 2.17±1.05%, Table S1) and more than double that of cackling geese (1.02±0.59%) and black brant (0.97%±0.79%).

**Table 2:** Seasonal variation in percent prevalence of LPAI viruses (% actively shedding) in four species of geese on the Yukon-Kuskokwim Delta, Alaska, 2006–2010.^1.^

Species	Spring Migration	Nesting	Molt	Total
Emperor goose	2.94^2^ (463)^3^	0.0 (459)	0.0 (1141)	0.82 (2063)
Greater white-fronted goose	2.17 (4209)	–	0.0 (149)	1.57 (4358)
Cackling/Canada goose	1.02 (2560)	–	0.0 (1570)	0.58 (4130)
Black brant	0.97 (844)	0.34 (596)	0.0 (2332)	0.24 (3772)
Total	1.78 (8076)	0.19 (1055)	0.0 (5192)	0.83 (14323)

1 Data from 2006 (with all 3 time periods combined), presented previously [14].

2 Grand mean.

3 Sample size.

**Table 3:** Comparison of candidate models for predicting prevalence of infection rates of low path AI viruses among four species of geese on the Yukon-Kuskokwim Delta, Alaska, 2006–2010.

Model	AIC	ΔAIC	No. of Parameters	AIC Weight
Species+Year	1257.6	0	8	0.620
Species w/unique quadratic effect of year for each species	1258.9	1.34	12	0.317
Species with quadratic effect of year	1262.2	4.60	6	0.062
Year	1277.7	20.16	5	0
Species	1392.1	134.54	4	0
Species, Linear effect of year	1393.9	136.33	5	0
Null Model	1415.0	157.38	1	0

Models with the lowest AIC values had the best fit.

### Seroprevalence

We tested a total of 541 adult geese for the prevalence of AI virus antibodies in sera in 2009 and 2010 (Table 4). The most competitive models explaining variation in seroprevalence were ones that included either Continental Affinity or Staging and Wintering Distribution (Table 5). Sex and Year also were relevant factors with summed AIC weights of 0.647 and 0.952, respectively. The inclusion of year in the top model is related to higher seroprevalence in 2010 compared to 2009, across all species and sexes (Table 3). Sex was also included in the top models, as seroprevalence was higher in males than in females for all species and years, except for emperor geese (Table 4). The greatest difference between sexes in seroprevalence was in 2010 for cackling geese (74.5% for males vs. 54.9% for females) and greater white-fronted geese (61.5% for males vs. 43.8% for females).

**Table 4:** Prevalence of AI antibodies in sera of adult geese on the Yukon-Delta, Alaska sampled during wing molt in 2009 and 2010 as determined from standard ELISA test.

Species of goose	2009 Males	2009 Females	2010 Males	2010 Females	Total Males	Total Females
Emperor	*n* = 32	*n* = 49	*n* = 13	*n* = 18	*n* = 45	*n* = 67
% Positive	87.5	100.0	100.0	100.0	91.1	100.0
95% CI^1^	(71.0–96.5)	(93.0–100)	(75.3–100)	(81.5–100)	(78.8–97.5)	(94.6–100)
Greater white-fronted	*n* = 17	*n* = 12	*n* = 13	*n* = 16	*n* = 30	*n* = 28
% Positive	52.9	33.3	61.5	43.8	56.7	39.3
95% CI	(27.8–77.0)	(9.9–65.1)	(31.6–86.1)	(19.8–70.1)	(37.4–74.5)	(21.5–59.4)
Cackling	*n* = 59	*n* = 37	*n* = 47	*n* = 51	*n* = 106	*n* = 88
% Positive	44.1	40.5	74.5	54.9	57.5	48.9
95% CI	(31.2–57.6)	(24.8–57.9)	(59.7–86.1)	(40.3–68.9)	(47.6–67.1)	(38.1–59.8)
Black brant	*n* = 29	*n* = 36	*n* = 41	*n* = 71	*n* = 70	*n* = 107
% Positive	48.3	38.9	51.2	45.1	50.0	43.0
95% CI	(29.4–67.5)	(23.1–56.5)	(35.1–67.1)	(33.2–57.3)	(37.8–62.2)	(33.5–52.9)
Total	*n* = 137	*n* = 134	*n* = 114	*n* = 156	*n* = 251	*n* = 290
% Positive	56.2	61.2	67.5	54.5	61.4	57.6
95% CI	(47.5–64.7)	(52.4–69.5)	(58.1–76.0)	(46.3–62.5)	(55.0–67.4)	(51.7–63.3)

1 Confidence interval.

**Table 5:** Comparison of best candidate models for predicting prevalence of antibodies to avian influenza viruses in four different species of geese on the Yukon-Kuskokwim Delta, Alaska, 2009–2010.

Model	AIC	ΔAIC	No. of Parameters	Model Likelihood	AIC Weight
Staging & Winter Distribution^1^+Sex+Year	626.61	0	5	1.000	0.260
Continental Affinity^2^+ Sex+Year	626.79	0.18	4	0.914	0.238
Staging & Winter Distribution+Year	627.64	1.03	4	0.598	0.155
Species+Sex+Year	628.15	1.54	6	0.463	0.121
Continental Affinity+Year	628.54	1.93	3	0.381	0.099
Species+Year	629.14	2.53	5	0.282	0.073
Continental Affinity+Sex	632.71	6.10	3	0.047	0.012
Continental Affinity	633.21	6.60	2	0.037	0.010
Staging & Winter Distribution	633.39	6.78	3	0.034	0.009
Staging & Winter Distribution+Sex	633.40	6.79	4	0.034	0.009
Species	634.88	8.27	4	0.016	0.004
Species+Sex	634.92	8.31	5	0.016	0.004
AI Virus Prevalence^3^+Sex+Year	635.44	8.83	4	0.000	0.000
AI Virus Prevalence+Year	636.45	9.84	3	0.000	0.000
AI Virus Prevalence	635.44	14.78	2	0.000	0.000
Social Index (nesting)^4^	717.09	88.48	2	0.000	0.000
Winter Habitat Use^5^	725.55	98.94	2	0.000	0.000
Null	733.02	106.41	1	0.000	0.000
Sex	734.27	107.66	2	0.000	0.000
Year	734.92	108.31	2	0.000	0.000
Sex+Year	736.07	109.46	3	0.000	0.000

Models with the lowest AIC values had the best fit. (Not all model results given for Social Index and Winter Habitat Use due to poor model fit).

1 Emperor geese winter in the Aleutian Islands and Alaska Peninsula, black brant on the Pacific Coast from British Columbia to Mexico, and cackling geese and greater white-fronted geese co-occur in interior valleys of Washington, Oregon, and California.

2 Cackling geese, greater white-fronted geese, and black brant (the latter with rare exception), spend their entire life cycle within the western hemisphere, whereas emperor geese are endemic to Alaska and the Bering Sea region and spend part of their life cycle in Asia.

3 Proportion of birds infected with AI viruses in spring (see Methods).

4 Black brant are colonial nesting while the other species are dispersed nesters.

5 Black brant and emperor geese winter in coastal marine environments, whereas cackling geese and greater white-fronted geese use agricultural areas.

The strength of the top fifteen models (ΔAIC<15.0; Table 5) is due almost solely to the high seroprevalence of emperor geese compared to the other species (96.5% seroprevalence overall versus <55% for the other 3 species; Table 4). Although AI virus prevalence was not included in the top model, models which included this variable still performed much better than the null model; an indication that this parameter was a good predictor variable. This model performed as well as it did because of the relatively high prevalence of AI viruses in emperor geese in spring. Indeed, two life history traits of the emperor goose (Continental Affinity and Staging and Winter Distribution), were each the strongest single covariate models (i.e. ΔAIC = 6.60 and 6.78, respectively; Table 5). Emperor geese are unique, as they are the only species in this study with a strong connectivity with Asia, and the only species that staged and wintered within Beringia (Aleutian Islands and on the Alaska Peninsula). Models with Social Index performed poorly, likely because black brant, the only colonial nesting species, had similar seroprevalence rates to dispersed nesting cackling geese, and greater white-fronted geese. Similarly, Winter Habitat Use was a poor predictor of seroprevalence. Both emperor geese and black brant rely heavily on marine resources during winter, but seroprevalence was much more common in emperor geese than black brant, the latter of which whose seroprevalence was similar to the two species (cackling geese and greater white-fronted geese) that primarily use agricultural lands when at southern latitudes (Table 5).

In 2009 all 3 goslings tested (one cackling goose and two emperor geese) were seropositive for AI viruses. However, in 2010, only 1 greater white-fronted gosling was seropositive out of 98 total samples (38 cackling geese, 31 emperor geese, and 29 greater white-fronted geese). Hence overall, seroprevalence in goslings was 4% (4/101). Fifty-three of the black brant sampled in 2009 and 2010 were of known age, as they were initially banded as goslings. For these individuals there was a positive relationship between age and presence of AI virus antibodies in their sera (Table 6). None of the 14 birds ≤3 years of age showed past exposure to AI viruses (95% CI: 0–0.232), whereas 19 of 39 (48.7%; CI: 0.324–0.652) of birds 4 years of age or older tested positive for AI virus antibodies (Fisher’s Exact Test; χ^2^_1_ = 10.63, *P* = 0.0011). The model that best explained the effect of age on seroprevalence in black brant was one which grouped birds into two age groups, <4 years of age, and ≥4 years of age (AIC = 58.04, versus AIC = 67.04 for a linear relationship, AIC = 63.88 for a quadratic [age+age^2^] relationship, and AIC = 71.17 for an intercept only model).

**Table 6:** Prevalence of AI antibodies in sera relative to age of black brant on the Yukon-Kuskokwim Delta, Alaska, 2009–2010.

Age at Capture	No. negative	No. positive	% Positive
1	5	0	0
2	2	0	0
3	7	0	0
4	7	4	36.4
5	5	6	54.6
6	3	2	40.0
≥7	5	7	58.3

There was no evidence of seroreversion during our study, although sample sizes were small. Of the 8 adult geese tested for seroprevalence in 2009 and recaptured in 2010, 6 that were seropositive in 2009 were also positive in 2010, one was negative in both years, and one that was negative in 2009 was positive in 2010.

## Discussion

### Interpretation of Serological Data

Seroprevalence is widely accepted as being reflective of viral exposure [32]. However, a variety of factors can influence population level estimates of seroprevalence, including the ability of individuals to seroconvert, antibody persistence, time since infection, and time interval between infection and sampling [40], [48], [49]. Fortunately, in our study we measured seroprevalence and AI virus infection rates over time. Longitudinal studies such as ours are able to account for, at least in part, the influence of persistence, and timing and magnitude of infection on seroprevalence rates [48], [49]. Also studies such as ours, with moderate to high levels of seroprevalence, are not as impacted by factors possibly affecting low population levels of seroprevalence, such as high mortality rates in infected birds, or a long time interval between infection and sampling [40].

### Influence of Ecology and Life History Characteristics on AI Virus Exposure

**Figure 4:**
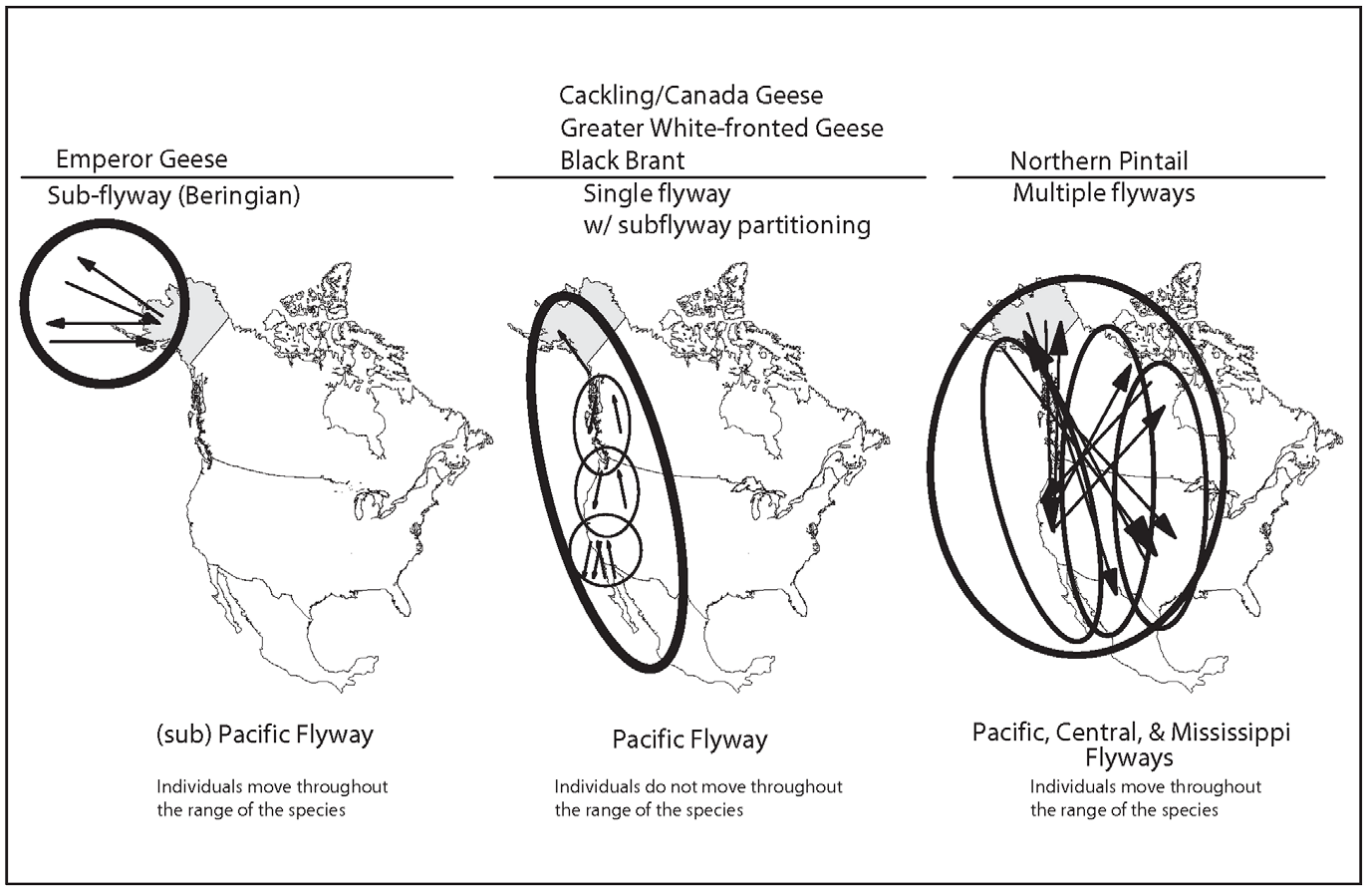
Diagrammatic examples of how viral transmission may be affected by species differences in distribution and population mixing.

Our finding that emperor geese had higher exposure rates than the other geese in our study may be more related to differences in distribution during the annual cycle compared to the other species rather than other specific life history characteristics (Table 1). Besides the aforementioned link with Asia, emperor geese have a more restricted overall distribution (one that is centered within Beringia - eastern Siberia to the Aleutian Islands in western Alaska, [50], [51]), than the other species in this study and as a result exhibit less population structuring than the other geese. This pattern may predispose emperor geese to be relatively uniformly exposed to AI viruses. In contrast, the other three species of geese are distributed throughout the Pacific Flyway, from Alaska to Mexico, and exhibit population structuring, with either recognized subspecies (cackling/Canada geese, greater white-fronted geese; [52], [53]) or other evidence of restricted gene flow [54], [55], [56]. Intra-species differences in nesting distribution and use of spatially or temporally discrete staging and wintering areas likely lead to species differences in virus transmission (Figure 4). Population size might also influence the prevalence and stability of AI viruses in host populations although it seems unlikely to contribute to the patterns we have shown in Y-K Delta geese, as population estimates of the number of nesting birds for the four species are of the same order of magnitude, ranging from 73,000 to 364,000 birds (5-year average, 2007–2011) [57].

The influence of life history characteristics on exposure to AI viruses can be subtle, especially across taxonomically similar species. More marked differences in behavioral and life history attributes should result in even greater intra-specific variation in AI exposure and pathogen prevalence. It is precisely this variation that may be contributing to the higher incidence of AI infection reported for ducks compared to geese [7], [14], [38], [39]. Ducks, especially dabbling ducks (Tribe Anatini), are more likely to disperse and often have broader distributions than geese. Indeed, even within the genus *Anas*, northern pintail ducks (*Anas acuta*) are more likely to disperse and have a broader range within, or across flyways than other dabbling ducks, such as mallards (*Anas platyrhynchos*), and this is reflected in patterns of AI virus gene flow and spread [58], [59], [60], [61] (Figure 4). Other life history attributes of geese that may limit intra-specific contact and minimize viral transfer compared to ducks include higher site fidelity (breeding and wintering), long term pair bonds, family cohesion, and assortative mating [52]. Geese also differ from ducks in terms of habitat use, as geese are less restricted to wetland habitats than ducks, and birds using wetland habitats are more likely to be infected with AI viruses [62].

Results from our study could be influenced by species differences in infection characteristics, especially the duration of viral shedding. Multi-species challenge studies could be very informative in this regard, especially if a variety of different LPAI subtypes were included to better mimic conditions in the wild.

### Influence of Age and Sex on Seroprevalence

Maternal transmission of antibodies to offspring in birds occurs via the egg [63]. The persistence of maternal antibodies in young birds varies across species, as antibodies are rarely detectable past 2 weeks of age in domestic poultry, but are still present up to 5 weeks of age in parrots (Family Psittidae), and gulls (Family Laridae) [64], and are thought to remain viable longer in large-bodied species. Our finding that only 4% of goslings (out of 101) tested positive for AI virus antibodies is similar to the 0% reported for 83 young pink-footed geese (*Anser brachyrhynchus*) sampled on Svalbard, Norway [21]. We could not determine whether goslings were seropositive due to retention of maternal antibodies or because of viral contact after hatching. However, the fact that no adult geese tested positive for AI viruses during the molt period would indicate that recent exposure was likely not the source of the antibodies in goslings. Since duration of retention of maternal antibodies is a function of antibody titers in the female at the time of egg laying [64], it could be that a few laying females had especially high levels of antibodies in 2009, the only year in which AI virus antibodies were detected in goslings.

The prevalence of AI virus antibodies in post fledging geese has also been shown to be related to age. In pink-footed geese, juveniles (birds in their first year of life) had a lower rate of seroprevalence than adult birds [21], which is to be expected as young birds generally have more limited exposure to pathogens than adults which have completed multiple migration cycles. However, even in adult birds, detectable antibodies only persisted for an average of 11 months in pink-footed geese, based on repeated samples of different wild birds over time (from spring to summer) [21]. Our finding that no black brant <4 years of age were seropositive indicates there may be a threshold age at which black brant form antibodies to AI viruses, or more likely, that birds <4 years of age are rarely exposed to AI viruses. The latter could be related to breeding experience, or age-specific differences in the use of molting or wintering areas by black brant from the Y-K Delta [55].

A higher exposure of males to AI viruses compared to females, as found in this study, is not surprising, as earlier research in Alaska on a larger number of species of migratory birds found that AI virus infection among Alaskan birds was higher in males than females [14]. Males may be more likely to be exposed to AI viruses in part because in waterfowl, females are philopatric and males disperse [65], with increased mobility likely leading to greater viral exposure. Sex differences in other behavioral attributes also likely influence virus exposure, as male geese defend females, nesting territories, and young during the summer and their mate and family during the winter, and hence likely have more intra – and inter specific interactions than females [29], [66]. Defense often includes fighting, which may entail hissing and biting – oral contact that is a recognized route of AI virus transmission [41]. In contrast, male pink-footed geese had lower AI virus exposure than females, although the difference was only significant during spring [21]. Differences between the study on pink-footed geese [21] and ours could be related to sex differences in population age structure, or increased physiologic stress on females preparing for migration and reproduction.

### Temporal Variation in AI Infection Rates and Seroprevalence

Our finding that AI virus infection varied across years has been commonly reported in multi-year studies. Indeed, several authors have reported a cyclic nature to the prevalence of AI viruses in waterfowl populations, with a peak every 2 to 4 years [67], [68], [69], [70]. In most of these studies the proportion of birds actively shedding viruses was generally highest during late summer and early autumn [7], [27]. It has also been found that high virus prevalence in autumn likely declines as migration proceeds, forming a north-south gradient of viral presence, with mallards sampled in Sweden exhibiting AI virus rates nearly twice that of mallards sampled in The Netherlands during the same time period [39]. This is opposite the trend found for greater white-fronted geese in The Netherlands [41], where geese returning from breeding areas showed very low AI virus prevalence. Greater white-fronted geese in the Pacific Flyway appear to have a similar seasonal pattern of infection, as no birds in our study tested positive for AI viruses during the late summer wing molt in Alaska, whereas 2.4%–5.9% of the same Pacific Flyway population of birds wintering in California were positive for AI viruses during over-lapping years of study [71] (for 2007–2008), and [72] (for 2005–2008). These values are similar to AI prevalence reported for greater white-fronted geese in Europe, where infection rates of over-wintering birds varied from 2.6 to 10.7% over 4 years of study [41].

Our best models explaining seroprevalence rates in geese included a year effect, and this was especially evident in cackling geese, which showed an increase in prevalence from 42.7% to 64.3% from 2009 to 2010. This follows the trend in AI virus prevalence, which was higher in spring harvested geese in 2010 than in 2009 (Figure 3). If AI virus antibodies are generally conserved in geese (see below), then seroprevalence would not necessarily be expected to track infection rates; the fact that such a trend is apparent for only one of the three species studied is of interest, and worthy of further investigation. Seasonal differences in antibody prevalence in pink-footed geese has been attributed to seroreversion [21]. Although the pink-footed goose study only encompassed one year [21], it is reasonable to expect that factors leading to seasonal variation in seroprevalence might also contribute to annual variation. We sampled few individual birds across time, so our findings are tentative, but the fact that none of the 8 individuals sampled in successive years seroreverted, and that age is positively correlated with antibody presence in black brant indicates that antibodies may be conserved, supports the contention that long-lived organisms are likely to have a strong humoral response [73].

### Chronology of Infection

Waterfowl infected with LPAI viruses generally shed viruses for <10 days, with geese shedding LPAI viruses longer than ducks [74]. However, interspecific differences in shedding characteristics are likely, and Canada geese have been reported to shed LPAI viruses for a maximum of 6 days [18], [75]. Given a relatively short period of infection, birds shedding AI viruses in Alaska during late April and early May likely were exposed to AI viruses on spring staging areas or shortly after arrival on the breeding area, as spring migration is thought to take 1–3 weeks in geese that winter south of Alaska (all species except emperor geese in this study). Even emperor geese do not arrive on breeding areas until almost 2 weeks after departing wintering areas in the Aleutian Islands and southern Alaska Peninsula [51]. Exposure to AI viruses is apparently quite rare while geese are nesting or raising goslings, based on the small proportion of geese that are actively shedding AI viruses during this time (Table S1).

### Relationship between Prevalence of AI Antibodies and Viruses

Until recently it was unclear whether waterfowl mounted a protective immune response when challenged by AI viruses, which in part explains why there have been so few serologic investigations of wild waterfowl and fewer still that report rates of both seroprevalence and AI virus infection [20], [24]. Seroprevalence studies of wild birds that have been conducted report a much higher proportion of birds exposed to AI viruses compared to AI virus infection rates of the same species. Indeed, a study in Italy found that up to 45% of wild ducks had been exposed to AI viruses despite only 1.5% of ducks testing positive for AI viruses [19], and antibodies to H5 and/or N2 AI virus subtypes were found in 34% of Canada geese in Pennsylvania, despite no birds testing positive for AI viruses [75]. A survey of 62 species of wild birds in North America found 25% to be positive for AI virus antibodies (including 23.6% of 106 Canada geese, and 45.6% of 329 wild dabbling ducks) [20]; values substantially higher than AI virus infection rates commonly reported for wild birds [7], [17]. The relatively low incidence of AI virus antibodies previously reported in Canada geese [20], [76], [77] compared to our findings may be related to lower AI virus exposure rates of Canada geese in mid-continent and southern U.S. than in Alaska. Many of the Canada geese from these regions are non-migratory [28], which likely reduces their encounter probability with birds carrying AI viruses [8]. The degree of correspondence between AI virus infection rates and seroprevalence is a function of many different factors including antibody persistence, duration of viral shedding (i.e. detection probability), timing of sampling, and factors potentially influencing immune response, such as age and condition. Our findings of seroprevalence rates of 47–96% across species despite viral shedding rates of only 0.15–2.28% is similar to that reported for pink-footed geese where 63% carried antibodies to AI viruses, but only 0.8% tested positive for AI viruses [21]. The latter study has been the most comprehensive to date and has revealed seasonal and age-related (adult vs. juvenile) variation in seroprevalence.

## Supporting Information

Table S1 Seasonal and annual variation in occurrence (% positive) of avian influenza viruses in four species of geese on the Yukon-Kuskokwim Delta, Alaska, 2006–20101.
(DOCX)
