# Supplementary material for: Evidence that Life History Characteristics of Wild Birds Influence Infection and Exposure to Influenza A Viruses
Source: PLoS One. 2013 Mar 4;8(3):e57614. doi: 10.1371/journal.pone.0057614 (PMC3587647; doi:10.1371/journal.pone.0057614)
Supplement: Table S1 — Seasonal and annual variation in occurrence (% positive) of avian influenza viruses in four species of geese on the Yukon-Kuskokwim Delta, Alaska, 2006–20101. (DOCX) [file pone.0057614.s001.docx]

Table S1. Seasonal and annual variation in occurrence (% positive) of avian influenza viruses in four species of geese on the Yukon-Kuskokwim Delta, Alaska, 2006–2010^1^.

|  | Spring Migration | | | | | | Nesting | | | | | | Molt | | | | | |
| --- | --- | --- | --- | --- | --- | --- | --- | --- | --- | --- | --- | --- | --- | --- | --- | --- | --- | --- |
| Goose Species | 2006 | 2007 | 2008 | 2009 | 2010 | Total^2^ | 2006 | 2007 | 2008 | 2009 | 2010 | Total | 2006 | 2007 | 2008 | 2009 | 2010 | Total |
| Emperor | 7.75 | 2.95 | 0.0  (0.025)^3^ | 0.0  (0.192) | 4.0 | 2.94+ 1.44 | 0.0 | 0.0 | 0.0 | 0.0 | 0.0 | 0.0 | 0.0 | 0.0 | 0.0 | 0.0 | 0.0 | 0.0 |
| *N* | 142 | 136 | 121 | 14 | 50 | 463 | 118 | 49 | 92 | 100 | 100 | 459 | 423 | 206 | 320 | 129 | 63 | 1141 |
| Greater white-fronted | 4.81 | 0.0  (0.005) | 0.11 | 1.40 | 4.52 | 2.17+ 1.05 | -- | -- | -- | -- | -- | -- | -- | 0.0 | 0.0 | 0.0 | 0.0 | 0.0 |
| *N* | 728 | 601 | 952 | 999 | 929 | 4209 |  |  |  |  |  |  |  | 29 | 31 | 30 | 59 | 149 |
| Cackling^4^ | 2.56 | 0.0  (0.007) | 0.0  (0.005) | 0.17 | 2.38 | 1.02 + 0.59 | -- | -- | -- | -- | -- | -- | 0.0 | 0.0 | 0.0 | 0.0 | 0.0 | 0.0 |
| *N* | 351 | 408 | 635 | 579 | 587 | 2560 |  |  |  |  |  |  | 216 | 271 | 462 | 251 | 370 | 1570 |
| Black brant | 4.08 | 0.0  (0.012) | 0.78 | 0.0  (0.027) | 0.0  (0.014) | 0.97 + 0.79 | 0.48 | 0.0 | 0.85 | 0.0 | 0.0 | 0.27 + 0.17 | 0.0 | 0.0 | 0.0 | 0.0 | 0.0 | 0.0 |
| *N* | 147 | 252 | 123 | 110 | 212 | 844 | 208 | 140 | 117 | 65 | 66 | 596 | 1094 | 551 | 495 | 79 | 113 | 2332 |

^1^Results from HEDDs data [33]; samples designated as positive for Original swab MA Call Positive (2006) and Summary MA Call Positive (2007–2010). Samples in 2006 were cloacal only, whereas in 2007–2010

cloacal and oral/pharyngeal samples were pooled. Data from 2006 initially presented for all of Alaska without information on season presented previously [14].

^2^Grand mean.

^3^Maximum undetectable prevalence [40].

^4^Spring samples may also include small-bodied Canada geese (*Branta canadensis subspp.)*.
